# Behavioural and Autonomic Regulation of Response to Sensory Stimuli among Children: A Systematic Review of Relationship and Methodology

**DOI:** 10.1155/2017/2629310

**Published:** 2017-11-28

**Authors:** Ivan Neil Gomez, Cynthia Y. Y. Lai, Paulin Grace Morato-Espino, Chetwyn C. H. Chan, Hector W. H. Tsang

**Affiliations:** ^1^Department of Rehabilitation Sciences, The Hong Kong Polytechnic University, Hung Hom, Kowloon, Hong Kong; ^2^Center for Health Research and Movement Science, College of Rehabilitation Sciences, University of Santo Tomas, Manila, Philippines

## Abstract

**Background:**

Previous studies have explored the correlates of behavioural and autonomic regulation of response to sensory stimuli in children; however, a comprehensive review of such relationship is lacking. This systematic review was performed to critically appraise the current evidence on such relationship and describe the methods used in these studies.

**Methods:**

Online databases were systematically searched for peer-reviewed, full-text articles in the English language between 1999 and 2016, initially screened by title and abstract, and appraised and synthesized by two independent review authors.

**Results:**

Fourteen Level III-3 cross-sectional studies were included for systematic review, among which six studies explored the relationship between behaviour and physiological regulation of responses to sensory stimuli. Three studies reported significant positive weak correlations among ASD children; however, no correlations were found in typically developing children. Methodological differences related to individual differences among participants, measures used, and varied laboratory experimental setting were noted.

**Conclusion:**

This review suggests inconclusive evidence supporting the relationship between behavioural and physiological regulation of responses to sensory stimuli among children. Methodological differences may likely have confounded the results of the current evidence. We present methodological recommendations to address this matter for future researches. This trial is registered with PROSPERO registration number CRD42016043887.

## 1. Introduction

Research concerning children's responses to sensory stimuli has been a growing field over the past few decades [1–3]. Variations in terminologies, conceptualizations, and definitions related to the responses to sensory stimuli have been observed [3]. Even with these broad definitions, several overarching features can be drawn from previous research related to children's responses to sensory stimuli. The relevant literature suggests that individuals' responses to sensory stimuli involve the following components: (1) reactivity; (2) sensory information input from the external environment; and (3) generation of a response [3–7]. Thus, in this review, the responses to sensory stimuli are operationally defined to be the child's reactivity to sensory information emanating from the external environment with subsequent responses. The regulation of these responses to sensory stimuli has been linked to adaptive behaviour necessary to participate in daily life activities, learning, and overall development [1, 8]. The incidence of difficulty in the regulation of responses to sensory stimuli has been suggested to be as much as 40–80% in children with disabilities and about 5–16.5% of their typically developing peers [4, 5, 9]. Given this prevalence and the resulting implications, there has been an increase in the literature to explore how responses to sensory stimuli are regulated among children [10]. The accumulating literature suggests two streams of thought exploring the regulation of responses to sensory stimuli: (1) outward behavioural data from parent/caregiver reports (i.e., parent reports and direct behavioural observations) and (2) physiological data from autonomic measures of the child's sensory arousal and reactivity (i.e., PNS and/or SNS measures). This paper reviews the extant literature that examines the relationship between these two streams of regulation and the methods related to a child's responses to sensory stimuli.

### 1.1. Behavioural Regulation of Responses to Stimuli

Sensory behaviour comprises observable outward reactions to external sensory stimuli, which may appear to be impaired among childhood clinical populations resulting in sensory symptoms or issues related to functioning in daily life [3, 4, 7]. Regulation of sensory behaviour may reflect how a child processes sensory information from his external environment in everyday situations and the subsequent outward behavioural responses [7, 11]. Children are constantly responding to environmental sensory stimuli, and in most cases these responses are adaptive. However, among those children who have issues in the behavioural regulation of responses to sensory stimuli, atypical responses to sensory input may occur and hamper the child's functioning in daily life activities [3, 7]. Sensory modulation disorders (SMD) reflect this atypical sensory behaviour to varying degrees and follow a conceptual nosology: overresponsive, underresponsive, and seeking/craving [12, 13]. A sensory overresponsive child may present with negative or aggressive behaviour in response to nonnoxious sensory information (i.e., anxiety when hearing the school bell, not liking to be touched, and dislike of certain food texture/taste). The sensory underresponsive child appears not to detect the sensory information (i.e., does not respond to their name when called and cannot recognize when their face or body is soiled). A child who is sensory craving/seeking needs unusually excessive amounts of sensory stimuli (i.e., incessant running/jumping around, mouthing of objects, and humming). It must be noted that these atypical responses to sensory stimuli are automatic and not willful [13].

The behavioural regulation of responses to sensory stimuli may be assessed using several measures including direct observation by professionals or parent-reported measures. In this review, it is our concern to synthesize behavioural data using these parent-reported measures. Parent-/caregiver-reported measures of outward behavioural performance in daily life activity contexts has long been used in examining sensory behavioural differences and may likewise reflect the regulation of such behaviour [7, 11]. Research that utilized these kinds of questionnaires has suggested individual differences in the behavioural regulation of responses to sensory stimuli related to diagnosis, developmental stages, gender, ethnicity, and the environment among others [14–16]. Perhaps the more apparent body of evidence comes from literature differentiating sensory behaviour and symptoms between typically developing children and clinical population groups [9, 17, 18]. However, previous researchers suggested that behavioural evidence (i.e., parent-/caregiver-reported measures) may not be as objective due to confounding parental factors [19–21]. While these measure may reflect some items related to the regulation of emotions and even physiological symptoms, they are framed from a perspective where sensory behaviour is reflected as reactions or responses to sensory stimuli in daily life activities. It has been suggested that direct laboratory observations, such as those offered by physiological outcomes, may be a more objective choice in understanding the regulation of responses to sensory stimuli [22, 23].

### 1.2. Physiological Regulation of Responses to Stimuli

Physiological responses are defined as deviations of physiological parameters from a control value in response to a discrete stimulus [24]. Physiological measures are most commonly assessed by indexing the activity of the central (CNS) and autonomic (ANS) nervous system [25]. The literature that examines the physiological regulation of responses to sensory stimuli has focused on autonomic measures of the parasympathetic (PNS) and sympathetic (SNS) activities [5]. In this paper, we concentrate on the ANS, specifically the physiological functions of PNS and SNS in relation to the regulation of responses to sensory stimuli. Physiological responses of a singular autonomic branch may result in an increase, decrease, or no change of the index in context. However, the physiological responses of the ANS may likewise be interpreted in terms of the dynamic interaction between its parasympathetic and sympathetic branches [26]. The use of a single autonomic measure has been a more prevalent choice. For instance, it was initially proposed that children with atypical sensory behaviour may present with an underresponsive or overresponsive SNS as measured by electrodermal activity (EDA) using a multisensory stimuli laboratory paradigm compared to typically developing children [27]. The sympathetic nervous system (SNS) has previously been implicated in the responses to sensory information. Consequently, other researchers suggested that it might be due to significantly lowered PNS functions that may explain this variability using cardiac vagal tone (CVT) measures using a similar multisensory stimuli laboratory paradigm [28]. The PNS has likewise been suggested to indicate the ability to regulate internal responses.

Aside from the evidence on the objectivity of autonomic measures in indexing the regulation of responses to sensory stimulation, it also offers practicality and efficiency. For example, a heart rate monitor attached to a chest strap may offer a more objective and noninvasive measure of heart rate variability (HRV) that conforms to both industry and research standards and can index PNS functions [26, 29]. Likewise, finger cuffs with specialized sensors may offer a similar objective measure of physiological responses that reflects the SNS activity through EDA [5, 23, 30, 31]. The objectivity, ease of use, in situ measurement, noninvasiveness, and efficiency of autonomic measures may prove to be most relevant when the subjects of interests are children. These methods and instrumentation have been shown to be useful for both typically developing children and children from clinical populations (i.e., ADHD, ASD, LD, and SPD).

The use of noninvasive, indirect, and objective measures of responses to sensory stimuli has been documented in research over the past few years [13]. Using autonomic measures of regulation of responses to sensory events within the child's environment provides a deeper insight into the mechanisms underlying the processing of sensory information. Thus, autonomic measures may serve as a good option in research and clinical inquiries that aim to understand the regulation of responses to sensory stimuli using laboratory paradigm experiments. However, mixed evidence has suggested group differences in psychophysiological functions between children from typically developing and clinical populations [2, 3]. While behavioural evidence supports differentiation of these two groups, the physiological evidence proves to be incongruent. Possibly, this may be related to methodological variations between these studies on their choice of measures, instrumentation, procedures, and laboratory paradigms. For instance, Lydon et al. [2] reviewed similar studies and noted several variations. For one, the authors noted the use of different physiological autonomic measures (i.e., EDA and heart rate) among similar studies. It was also suggested that there is a differing index of specific measure components (i.e., EDA amplitude, magnitude, onset, and latency). Furthermore, there are some variations in the use of novel laboratory paradigms (i.e., Sensory Challenge Protocol and multisensory paradigm) and the experimental conditions (i.e., rest, stimulation, and recovery; stimulation only; rest and stimulation only) measured within such paradigms. Thus, when the degree of relationship between behavioural and physiological regulation of responses is tested or previous results are attempted to be recreated, the results may turn out to be unclear and need to be further examined.

### 1.3. Relationship between Behavioural and Physiological Regulation of Responses to Sensory Stimuli

McEwen [25] proposed that physiological responses and behavioural responses have interacting influences on each other, along with children's individual differences. As previously mentioned, since issues on atypical sensory behaviour are not under conscious will, physiological mechanisms may be involved as underlying explanations. Literature from the neuroscience field that investigates the relationships between behavioural and physiological regulation of responses to sensory stimuli has been elusive regarding conclusive recommendations [2]. Previous reviews encompassed attempts to corroborate clinical and neuroscientific research suggesting underlying neural features of sensory behaviour and symptoms and critically examined the literature on basic neurosciences to identify evidence of an underlying mechanism that explains sensory behaviour and symptoms and as it relates to sensory-driven neuroplasticity [4, 13]. Schauder and Bennetto [3] implied that, among children, specifically those diagnosed with ASD, research has been dichotomized into two different perspectives: (1) behavioural (sensory symptoms that are manifested as the child interacts with environmental sensory information in real life) and (2) neural (examining neural mechanisms and pathways underlying the physiological regulation of responses to sensory stimuli using laboratory paradigms). While these preceding studies can provide insight into the possible relationship between behavioural and physiological regulation, several caveats are noted. For instance, the authors' discussions mainly centred on CNS physiological substrates, offering limited acumen to peripheral autonomic measures. Moreover, there was limited salient evidence on the relationship between behavioural and physiological evidence on the regulation of responses to sensory stimuli.

In an attempt to resolve the dearth of associations between behavioural and physiological regulation of responses to sensory stimuli, Lydon et al. [2] synthesized results of 57 studies on behavioural and physiological reactivity found in ASD children. In a subsection of their review, the group reviewed four studies [5, 27, 31, 32] with inconclusive relationships between sensory behaviour and various physiological indices. The foregoing was an initial attempt to characterize such relationship through evidence synthesis from empirical studies; however, the synthesis and discussions were found to be limited. First, the studies included were specific to ASD and did not extend to expound on typically developing children or other clinical groups. Second, the reviewed evidence presented published data only until the year 2014. Thirdly, behavioural measures covered several outcomes other than sensory behaviour. Fourth, physiological measures focused mainly on CNS measures (i.e., electroencephalogram and cortisol), without succinctly covering physiological autonomic measures. Lastly, the review did not summarize the specific methods employed in the reviewed articles. The heterogeneous variations in the methods, whether behavioural or physiological, across research might further complicate the understanding of the evidence. Thus, other than systematically reviewing the evidence of relationships, there might be value in reviewing and synthesizing the methods of such researches to inform future inquiries on the topic or even to critically examine the rationale behind the choices that may provide insight into its possible influence on the results. The authors of this review hypothesize that variations in the methodologies could have played an indirect role in the inconsistencies of the findings. Taken together, there is a need to examine the existing evidence on the relationship and the associated methods of investigation between behavioural and physiological regulation of responses to sensory information among childhood populations.

As described previously and elsewhere, the evidence on the relationship between behavioural and physiological regulation of responses to sensory stimuli among children remains inconclusive. Furthermore, specific methodologies related to pertinent research inquiries are yet to be summarized and recommended. In response, we sought to examine the extant literature to systematically review and critically appraise the current evidence on the relationship between behavioural and physiological regulation of responses to sensory stimuli among children and describe the methods used in these studies. Specifically, this systematic review aims to answer the following research questions:

1. What is the evidence on the relationship between behavioural and physiological regulation of responses to sensory stimuli among children?
2. What are the methods used in investigating the behavioural and physiological regulation of responses to sensory stimuli research?

## 2. Materials and Methods

This systematic review aimed to synthesize the relationship and its pertinent investigative methods between behavioural and physiological regulation of responses to sensory stimuli among children. This review is registered on the PROSPERO database with registration number CRD42016043887 and is reported based on the Preferred Reporting Items for Systematic Reviews and Meta-Analyses (PRISMA) guidance (see Supplementary Material available online at https://doi.org/10.1155/2017/2629310) [33].

### 2.1. Study Selection

This systematic review considered studies for inclusion based on the criteria described below.

#### 2.1.1. Types of Studies

In this review, we considered both experimental and descriptive epidemiological study designs including descriptive cross-sectional studies for inclusion. Studies must have used either a correlational or between-group design.

#### 2.1.2. Types of Participants and Exposures

This review considered study participants between the ages 3 and 18 years from normative and clinical populations (i.e., autism, ASD, attention-deficit hyperactivity disorder, and sensory processing disorder). The age range was based on an initial scoping of the literature and report on the PROSPERO trial registry. However, due to limited papers for review during our actual systematic review, we reconsidered papers with participants within the range of 2–19 and reported the implications for the findings. Participants must have been assessed systematically to rule out/confirm sensory behavioural problems/difficulties and clinical diagnosis. Whenever possible, we report on the specific atypical sensory behaviour described in the reviewed articles.

#### 2.1.3. Types of Outcomes

We considered studies that included the following outcome measures: (1) behavioural measures (parent-/caregiver-reported measures) and (2) physiological measures, related to the regulation of responses to sensory stimuli. The physiological measure should be an ANS index. The laboratory paradigm should clearly indicate in its design the types of condition(s) when physiological responses were measured. These conditions (i.e., resting baseline, stimulation, and recovery) were flagged for further evaluation and consequent inclusion in the evidence synthesis and analysis.

#### 2.1.4. Literature Search

**Figure 1:**
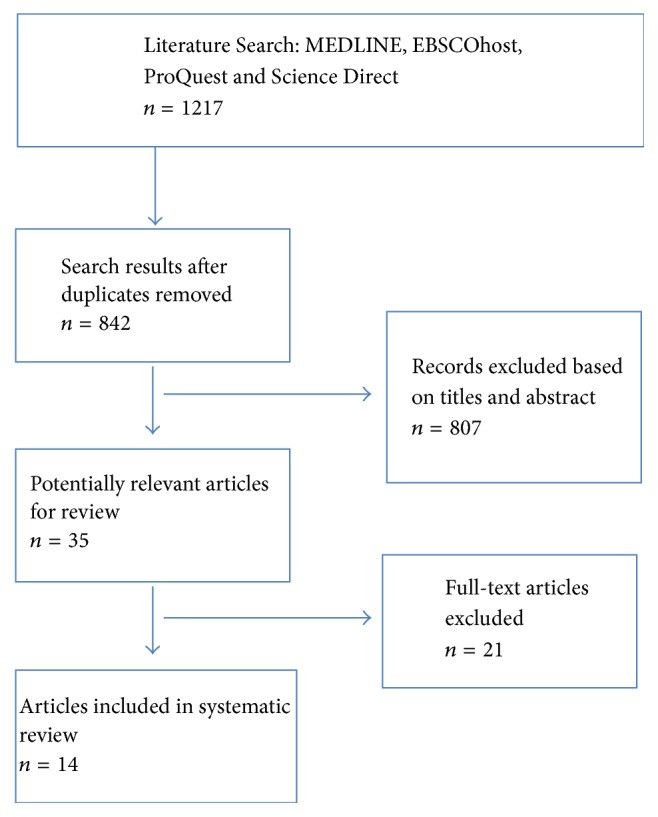
PRISMA flow diagram for studies from the systematic review.

Five electronic databases (Medline, EBSCOhost, ProQuest, Science Direct, and BioMed Central) were searched for peer-reviewed English language (or those with English translations) articles between January 1999 and December 2016. The search strategy included using the following terms validated by an experienced health science librarian: (1) Autonomic^*∗*^* or* ANS* or* PNS* or* SNS; (2) Sensory^*∗*^* or* SI* or* SPD, and multiple combinations between search terms. This review considered studies that included participants between the ages of 2 and 19 years from normative and clinical populations. The use of “*∗*” represents truncated terms implemented to increase the scope of the search for articles. The initial search tier yielded 1,217 articles, and after duplicates were removed, 842 articles were recorded. Title and abstract reviews further delimited the articles thereby excluding 807 articles. With the remaining 35 potentially relevant articles, 21 were excluded based on irrelevance to the research questions and scope of this systematic review, leaving 14 articles for systematic review by the authors (Figure 1).

#### 2.1.5. Appraisal, Extraction, and Analysis of Data

Fourteen articles were subjected to appraisal, level of evidence assessment, risk of bias assessment, data extraction, and analysis by two study authors (Ivan Neil Gomez and Paulin Grace Morato-Espino). When a disagreement occurred, a meeting was held for discussion to reach a consensus; when a consensus could not be reached, an independent third reviewer was consulted. Appraisal of articles and data extraction was performed using the Joanna Briggs Institute Meta-Analysis of Statistics Assessment and Review Instrument (JBI-MAStARI) [34] for quantitative studies. The data extracted included specific details about the populations, study methods, and results of significance to the review question and specific objectives. The evidence hierarchy classification of the National Health and Medical Research Council [35] was used in appraising the level of evidence of each screened article. We assessed the risk of bias within studies using the Cochrane Risk of Bias (RoB) tool [36]. Due to the heterogeneity of the outcome measures used and the focus on methodological aspects reported, it was deemed appropriate to settle on a systematic review article. Since statistical pooling was not possible, the findings are presented in a narrative form including tables to aid in data presentation. This involved the aggregation or synthesis of conclusions to generate a set of statements that represent aggregation, through assembling and categorizing these conclusions based on similarity of meaning.

## 3. Results

### 3.1. Appraisal of Study Quality, Level of Evidence, and Risk of Bias

Fourteen articles were subjected to appraisal of quality, levels of evidence, and risk of bias. Overall, the studies presented with moderately good quality after appraisal. These 14 articles were assessed to be Level III-3 cross-sectional studies. Across the studies, issues of reporting, performance, and detection bias ranged from probably low to high risk. Further information on these is reported in the appended Supplementary Material.

### 3.2. Study Characteristics

Fourteen Level III-3 cross-sectional studies were reviewed as part of the final analysis of this systematic review [5, 27, 28, 31, 32, 37–45].  Table 1 presents a summary of the 14 studies reviewed. The total sample size for the reviewed papers amounted to *n* = 1,085 participants between the ages of 2 and 19 years. From the total sample, *n* = 454 belonged to a typically developing (TD) group while the remaining comprised clinical populations: ASD = 373; attention-deficit hyperactivity disorder (ADHD) = 105; and sensory modulation disorder (SMD) = 153. It is worth noting that, in some studies, groups were further classified as TD with SMD = 9; ADHD with SMD = 21 [38]; and dual-diagnosis of ADHD and SMD = 12 [42]. All papers used at least one sensory behavioural outcome measure and one autonomic physiological measure.

### 3.3. Behavioural and Physiological Differences in the Regulation of Sensory Responses

There is a general trend in the stability of results suggesting sensory behaviour differences between TD children and children with (1) ASD [5, 31, 32, 37, 40, 41]; (2) ADHD [38, 39, 42]; and (3) SMD [5, 27, 28, 32, 44, 45]. However, there were variations in the results of autonomic physiological measures ranging from significant differences to none when clinical groups were compared to at least the TD children (see Table 1). Significant physiological differences were suggested by four studies: lower resting PNS in ASD and severe SMD [40]; lower PNS reactivity in SMD [28, 43]; and higher resting SNS reactivity in ADHD [31]. Ten studies, while reporting differences or atypical physiological responses to sensory stimuli, failed to reach significant levels [5, 27, 32, 37–39, 41, 42, 44, 45].

### 3.4. Behavioural and Physiological Relationship in the Regulation of Sensory Responses

Whereas all studies reviewed in this article reported similar methodologies related to behavioural and physiological regulation of responses to sensory stimuli, less than half performed correlational analysis (see Table 1). Six studies explored the relationship between behaviour and physiological regulation of responses to sensory stimuli [5, 31, 32, 37, 40, 41]. In these studies, separate correlational analyses were performed between TD and clinical groups of children. Clinical group data from all studies were from children with ASD, and the results suggest that adaptive sensory behaviour (i.e., child almost/always does not display atypical sensory behaviour) are significantly positive in correlation with resting PNS activity [40] and SNS reactivity upon sensory stimuli presentation [32, 37]. Interestingly, one study suggested otherwise and found that problems related to sensory behaviour are significantly related to greater SNS activity at rest and upon sensory stimulation [31]. Nevertheless, two studies reported no significant correlations between behaviour and physiological regulation of responses to sensory stimuli in ASD [5, 41] and SMD [5] on SNS measures at resting and reactivity conditions. No significant correlations were found for TD groups [5, 31, 32, 37, 40, 41]. However, interesting insights were offered by several authors on sensory-related behavioural issues possibly related to elevated resting PNS [31, 40] and elevated [31, 37] or depressed [32] SNS reactivity; yet the results did not yield significant thresholds nor reach moderate correlations.

### 3.5. Summary of Methods in the Reviewed Studies

#### 3.5.1. Behavioural Measures

We extracted information pertaining to the respondent (i.e., parent/caregiver reports of children's outward behaviour) reported measures on the sensory behaviours of children. Nine studies [5, 27, 28, 39–44] of the 14 reviewed articles used the Short Sensory Profile (SSP) [46] or its other versions (see Table 1). Matsushima et al. [40] used the Japanese version [47], while McIntosh et al. [27] used the original 51-item version; the rest opted to utilize the current 38-item parent/caregiver questionnaire. Other measures reported included Sensory Processing Measure (SPM) [48] used by Chang et al. [31]; Sensory Profile [7] used by Daluwatte et al. [37] and Su et al. [45]; Sensory Overresponsivity Scale [49] used by Lane et al. [38]; and the Evaluation of Sensory Processing [50] used by Su et al. [45].

#### 3.5.2. Autonomic Physiological Measures

In this review, we found eight studies using measures of electrodermal activity (EDA) to index functions of SNS activity [5, 27, 31, 37, 39, 41, 44, 45], of which only three used the specific component of skin conductance level (SCL) [5, 38, 41]. Three studies used indices of PNS functions, particularly heart rate variability-normalized unit of high-frequency bands (HRV-HF n.u.) [41] and cardiac vagal tone (CVT) [28, 43]. Intriguingly, one study by Schaaf et al. [44] simultaneously indexed both PNS and SNS functions of a dually innervated organ (i.e., heart) using respiratory sinus arrhythmia (RSA) and preejection period (PEP), respectively. The remaining two studies used nonspecific autonomic branch measures from different dually innervated organs subserved by the ANS: pupillary light reflex (PLR) [37] and heart rate (HR) [32]. These specific measures are further reported and summarized in Table 1.

#### 3.5.3. Laboratory Paradigm

Autonomic physiological measures are typically gauged within the context of a controlled laboratory paradigm. Among the reviewed articles, ten of the 14 studies reviewed [5, 27, 28, 31, 38, 39, 42–45] used the Sensory Challenge Protocol (SCP), a multisensory laboratory paradigm designed to present various stimulation conditions which was earlier described in the literature by Miller et al. [51].  Table 2 summarizes the variations in the SCP across the reviewed studies. Five of these studies followed exactly the original protocol [5, 27, 28, 39, 45] of which two studies [27, 39] implemented the original stimulation presentation schedules. Six studies [5, 27, 31, 42–44] included at least one additional sensory domain (i.e., additional auditory tones). One study reduced the number of trials from ten to eight [45]. The interstimulus interval (ISI) varied from 10 to 19 seconds, presented at pseudorandom or variable intervals. Four studies used different laboratory paradigms [32, 37, 40, 41], three of which developed their own paradigms based on the SCP [32, 40, 41], while one study [37] was specific to the autonomic physiological outcome of PLR and hence used only visual stimuli (i.e., light). With reference to event conditions in the paradigm, all the reviewed articles included at least a stimulation condition. However, several variations were noted. Ten studies added a resting baseline condition measured prior to the presentation of the sensory stimuli [5, 28, 31, 32, 37, 38, 40, 41, 43, 44]. Eight of the reviewed studies [5, 31, 37, 38, 40, 41, 43, 44] likewise included a recovery condition measured after the stimulation block, with contexts similar to the resting baseline condition.

## 4. Discussion

This systematic review identified 14 Level III-3 cross-sectional studies with similar methods that looked at similar methodologies related to behavioural and physiological regulation of responses to sensory stimuli in children. However, after streamlining specific studies that utilized correlational analysis, only six among these studies explicitly explored the correlation between behavioural and physiological regulation of responses to sensory stimuli in children. We found fairly consistent trends in the differences in behaviour and physiological regulation of responses to sensory stimuli between TD and clinical groups of children. Our findings support the concept of allostasis by McEwen [25] which suggests that individual differences may influence behavioural and physiological responses. However, other than medical or clinical diagnosis, certain personal factors may likely have influenced the evidence summary. Developmental differences in the regulation of responses to sensory stimuli have been previously proposed by other researchers [3, 41]. The wide age range (2–19 years) in the reviewed articles may possibly have influenced the context of our findings (i.e., growth and/or hormonal development). While the authors wanted to control for age, the limited available literature prevented this. Such age variations among other individual difference factors may have biased the results; hence behaviour and physiological regulation of responses to sensory stimuli are not always congruent [37, 41]. Future studies may need to consider other factors related to individual differences among children within their research designs.

### 4.1. Evidence on the Relationship between Behavioural and Physiologic Regulation of Responses to Sensory Stimuli

This systematic review identified three Level III-3 cross-sectional studies suggesting that adaptive sensory behaviour (i.e., child almost/always does not display atypical sensory behaviour) is related to increased autonomic physiological activity [32, 37, 40]. However, it was likewise discovered that sensory behavioural problems may also be related to the same increased sympathetic regulation [31] among the ASD group. This relationship suggests physiological evidence underpinning atypical sensory behaviour among ASD, which is a novel insight into the underlying aetiology of Sensory Processing Disorders [3]. In contrast, two Level III-3 cross-sectional studies concluded that behaviour and physiological regulation of responses are not related in ASD [5, 41], which parallels the results for typically developing children across all the reviewed studies that performed correlational inquiries.

Behavioural patterns in the regulation of responses to sensory stimuli may not be similar within and between groups of children. Miller et al. [12, 13] suggested that atypical sensory behaviour may present in several ways (i.e., overresponsive, underresponsive, or seeking/craving). Among the six reviewed studies, only one explicitly reported the specific pattern of such atypical sensory behaviour [5]. The other five studies [31, 32, 37, 40, 41] recruited ASD group populations. A prevalence rate of 90% of children with ASD was suggested to have some form of sensory issues [4, 5, 9], making this particular group a good representative of atypical sensory behaviour. However atypical sensory behaviour may also be seen in other clinical groups and even in TD populations of children. Schoen et al. [5], the only study that recruited children with specific issues related to modulation of sensory information, looked at the relationship between behavioural and physiological regulation of responses to sensory stimuli among ASD, TD, and SMD groups. Conversely, there was not enough published information as to the specific subtype of SMD. SMD represents a pattern of symptomology presentation under Sensory Processing Disorders (SPD) [12, 13]. The aforementioned three subtypes of atypical sensory behaviour fall under the pattern of SMD. Such nosology of sensory behaviour response modulation presentations may have been overlooked by the authors in the reviewed studies. At the other end of the spectrum though, it could also be that physiological responses follow a similar nosology of response patterns that is yet to be further understood [5]. The evidence summarized in this review on the relationship between behavioural and physiological regulation of responses to sensory stimuli among children is limited to children presenting with atypical sensory behaviour, specifically in ASD groups. Our findings seem to recommend that behavioural and physiologic responses possibly represent two different streams of regulation, whose individual mechanisms need to be further explored. Furthermore, there seems to be a dearth of literature examining the same correlational hypothesis among other clinical groups known to have atypical sensory behaviour (i.e., ADHD and LD). It is worth mentioning the limited inquiry into the correlation between behavioural and physiological regulation of responses to sensory stimuli in children. While all articles reviewed in this paper used both behavioural and physiological measures, less than half only explored the correlation between the two. Future research utilizing both measures needs to further establish whether such correlation does exist between behavioural and physiological regulation of responses to sensory stimuli in children. Correlational analysis between behavioural and physiological measures of regulation of responses to sensory stimulation in children may provide supporting evidence on the probable effect of neurophysiologic states on sensory behaviours. The authors further suggest testing the same hypothesis among these groups, with emphasis on the specific subtype of atypical sensory behaviour.

### 4.2. Methodological Variations in the Relationship between Behavioural and Physiological Regulation of Responses to Sensory Stimuli

A possible reason for the inconsistent findings on the relationship between behavioural and physiological regulation of responses to sensory stimuli revealed in this paper is attributable to variations in the method of investigation among the reviewed studies. Based on our review, it seems that there are three major ways in which the methodologies in these studies vary: individual differences (i.e., age and types of participants), measures used (i.e., behavioural and autonomic), and laboratory experimental setting (i.e., experimental paradigm and procedural/environmental control). As previously mentioned, individual differences related to age incompatibility may have influenced the results of this review. Among the six reviewed studies, three studies recruited school-aged participants [5, 31, 40], two studies recruited participants in early childhood [32, 41], and two studies recruited participants in their adolescence [5, 37]. Developmental variations, especially among the adolescent group, may have influenced the ANS by way of hormonal changes related to puberty [52, 53]. While the specific subtype of atypical sensory behaviour was not reported across all six studies, only one study was found to have assessed for SMD [5]. Besides variations in the demographic characteristics of the participants, there were variations in the behavioural measure used in these studies. Three studies used the Short Sensory Profile [5, 40, 41] and the rest used the Sensory Processing Measure [31] or variations of the Sensory Profile (i.e., Original Caregiver version [37]; Japanese version [40]; Infant/Toddler [32]). Variations in behavioural outcome measure would have resulted in differences in the test construct, psychometric properties of the measures (i.e., factor loading, subsection reporting, and response scales), and hence the scores on the measures. These would have influenced the results yielded for the behavioural variables for establishing their relationships with the physiological responses. The autonomic measures used among the studies likewise varied with three studies indexing the SNS using EDA [5, 31, 41], one study indexing the PNS using HRV [40], and two studies indexing dually innervated (PNS and SNS) organs using PLR and HR [32, 37]. Even when the same autonomic measure was used, specific parameters and its operational definition appear to vary across the studies. For example, of the three studies that used EDA as the physiological autonomic measure, only one component was found to be similar across (i.e., EDA magnitude) and other EDA components differed. While autonomic measures may be a good index of physiological responses, each would have a function specific to a related behavioural response that needs to be considered in future research designs [28, 39, 54]. The choice of experimental laboratory paradigm to elicit physiological responses poses a great influence on the methodological robustness and eventual uniformity and generalizability of findings across the studies [54]. Two of the reviewed studies [5, 31] used the Sensory Challenge Protocol, while the rest used their own novel sensory paradigm. The variations in the methodologies of the reviewed articles are important aspects in consideration of synthesis and analysis of the results prior to conclusive reporting of the evidence. In this review, while some evidence was found that may support the relationship between behavioural and physiological regulation of responses to sensory stimuli in children, the varying methods employed in the individual research may contribute to the fact that such relationship remains incongruent and inconsistent.

### 4.3. Methodological Caveats Related to Behavioural and Physiological Regulation of Responses to Sensory Stimuli

Methodological caveats were noted not only in the six studies that explored the relationship between behavioural and physiological regulation of response to sensory stimuli in children, but also in the other eight studies. We summarized and discussed these caveats and provide some recommendations. This systematic review found three main methodological caveats related to use of behavioural measures, physiological autonomic measures, and laboratory paradigm.

It must be noted that while the behavioural outcome measures used in the reviewed studies were developed to ascertain observable sensory behaviour, the use of these parent/caregiver-answered questionnaires could have likewise influenced the results. Since the reviewed articles mainly focused on the total scores from these measures, the resulting latent factor may have over-/underestimated subscales and/or factors in which problems could have been demonstrated. Behavioural measures may not be as objective and the information may be confounded by parental factors [19, 20, 55]. Furthermore, items on these behavioural measures might not reflect physiological symptoms related to the regulation of responses to sensory stimuli [16]. While useful in identifying how sensory symptoms affect daily living skills, it is unclear whether the latent factors in these questionnaires reflect constructs of physiological symptomology related to autonomic physiological functions. It may be interesting to use autonomic regulation symptom items within such sensory behaviour questionnaires such as those incorporated into the Sensory Processing and Self-Regulation Checklist [56] or the Sense and Self-Regulation Checklist [57]. Nevertheless, researchers are recommended to test these tools in future research.

The Sensory Challenge Protocol (SCP) initially described in the literature by Miller et al. [51] has been a popular laboratory paradigm used in the reviewed articles to elicit physiological responses to sensory stimuli. It is comprised of several stimuli under five sensory domains.  Table 3 provides a salient overview of the paradigm. SCP has long been referred to due to its ability to reliably quantify physiological regulation of responses to sensory stimuli [54]. SCP was developed to test multisensorial domain regulation of sensory responses and has been used alongside physiological autonomic measures (i.e., EDA, HRV, CVT, and RSA) [54]. However, the use of a multidomain sensory paradigm may not always be suitable. A recent literature review urges the use of modality-specific measurements to improve the sensitivity of measures [3]. The auditory modality seems to offer a more sensitive stimulus to elicit physiological responses [3, 31, 40, 41, 52]. In any case, studies that refer to SCP must modify with caution and decisions must be rationale-driven, supported by evidence from the physiological literature. Additionally, autonomic activity is influenced by external environmental factors related to temperature, humidity, and noise level among others [30, 52, 53, 58]. These factors should be discretely controlled during the laboratory paradigm experiment and explicitly reported.

Most of the reviewed articles employed the use of single autonomic physiological measures that may not fully represent the complex nature of the ANS. Of interest is the popularity of using EDA and its components. EDA is a widely used sensitive indicator of pure SNS activity [30, 52, 59] and has been used in identifying physiological activities related to sensory stimuli responses. However, the use of a single physiological measure should be approached carefully. The dynamic range of the physiological response of the ANS is determined by the interactive relationship of its branches [58]. Autonomic reciprocity as employed by indexing a singular physiological measure of the ANS is overly restrictive and unable to capture the complex control of the ANS. The dynamic interaction among various subsystems within the autonomic space mediates physiological homeostasis in response to external challenges [25]. Thus, researchers need to refocus the choice of autonomic measure to represent the allostatic relationship between the PNS and SNS [44]. However, we are further reminded to be thoughtful in choosing physiological measures from dually innervated organs (i.e., heart).

Heart rate variability (HRV) has been considered as a promising marker for autonomic activity [53, 60]. HRV has been an increasing outcome option in providing insight regarding individual differences in autonomic responses [58]. HRV indices of low-frequency (LF) and high-frequency (HF) bands represent the activity of SNS and PNS, respectively. Nevertheless, it has been argued whether the value of LF is a pure measure of SNS functions [61]. The group of Schaaf [28, 43, 44] has suggested the use of PNS measures, specifically CVT as indexed by RSA, which is a high-frequency component of HRV fluctuations that are in phase with inhalation and exhalation [62]. RSA is determined largely by vagal influences on the heart [62] but is greatly dependent on respiratory frequency and depth of ventilation [29]. While quantification for RSA can be performed by way of automatic algorithmic equations using sophisticated computational software, controlling for these factors traditionally depends on the use of pharmacological blockades and presents several impending ethical issues in paediatric research. Several recommendations have been suggested for controlling or indexing respiratory cycles using metronomes or respiratory monitors [29, 62]. However, this may pose additional internal and external challenges to the child that may consequently influence its physiological responses. Thus, its use may present methodological issues related to the invasiveness, efficiency, and measurement when performed among children. Previously, it has long been suggested to use independent measures to exemplify the dynamic interactions between the activities of PNS and SNS, ideally within the same organ or functional units [60]. Since HRV can measure both SNS (i.e., HRV-LF) and PNS (i.e., HRV-HF) simultaneously, future researchers may explore this option. However, the ability of the HRV-LF to index purely SNS functions remain inconclusive. Therefore, other than HRV, EDA can also be a supplementary measurement in this proposed research. Future researchers may explore the option of using the HRV-HF, HRV-LF, and EDA as multivariate measures of physiological response regulation.

While this review has provided several interesting results and arguments, several important limitations must be considered relevant to the actual quality of the reviewed articles. Aside from those previously discussed, the authors considered the methodological issues of the reviewed studies as reflected by the appraisal of quality, evidence, and risk of bias (see Supplementary Material). Most of the reviewed articles had issues on sample representativeness, unequal sample size, and the use of nonprobabilistic sampling methods. We also found some challenges on selection and reporting bias. While we recognize the implications of quality and bias on the results, the articles were still included due to limited available evidence on the topic. Caution is recommended in overinterpreting the results of this review. We recommend that researchers in the future develop their methods and protocols with these limitations in mind to improve the quality of the evidence generated.

## 5. Conclusions

This systematic review concludes that, up to date, there is inconclusive evidence that supports the relationship between behavioural and physiological regulation of responses to sensory stimuli in children. The reviewed methods in conducting behavioural and physiological regulation of responses to sensory stimuli in children are described and researchers are advised to critically examine the utility of these methods prior to adaptation and/or modifications in measures, instruments, paradigm, design, or setting. Methodological differences may likely have confounded the results of the current evidence. Future related research may benefit from improving the research designs through individual differences (i.e., age and types of participants) stratification and control, use of behavioural measures with autonomic regulation items, multiphysiological autonomic measures, and the use of a representative sensory stimuli (i.e., auditory). Nevertheless, our results provide some evidence that supports how physiological processes may relate to sensory behaviour, which may provide supporting neurophysiological evidence on the mechanism underlying atypical sensory behaviour as seen in some clinical populations, specifically in ASD. Furthermore, our review of the evidence recommends the development and use of behavioural measures related to the physiological regulation of sensory stimuli whose items reflect autonomic symptoms in daily living situations. The authors surmise that the incongruence in the existing body of evidence can be improved through salient considerations and empirical-driven choices in the methodologies of future researchers.

## Supplementary Material

Supplemental File 1: PRISMA Checklist.
Supplemental File 2: Summary of Appraisal, Level of Evidence and Risk of Bias.
Supplemental File 3: Summary of Risk of Bias in Individual Studies.
Supplemental File 4: Summary of Risk of Bias Across Studies.

## Figures and Tables

**(a):** 

Author	Year	Sample size	Age (yr)	Sample
Chang et al.	2012	50	5–12	ASD: 25; TD: 25^*∗*^
Daluwatte et al.	2015	259	5–19	ASD: 152; TD: 107^*∗*^
Lane et al.	2010	85	6–12	TD: 36; TDs: 9; ADHDt: 18; ADHDs: 21^*∗∗*^
Mangeot et al.	2001	56	5–13	ADHD: 26; TD: 30^*∗*^
Matsushima et al.	2016	69	6–12	ASD: 37; TD: 32^*∗*^
McCormick et al.	2014	87	2–4	ASD: 54; TD: 33^*∗*^
McIntosh et al.	1999	38	3–9	SMD: 19; TD: 19^*∗∗∗*^
Miller et al.	2012	176	6–12	SMD: 37; ADHD: 28; SMD/ADHD: 12;
				TD: 30^*∗∗∗*^
Schaaf et al.	2003	15	4–8	SMD: 9; TD: 6^*∗∗∗*^
Schaaf et al.	2010	83	5–12	SMD: 43; TD: 40^*∗∗∗∗*^
Schaaf et al.	2015	88	6–9	ASD: 59; TD: 29^*∗*^
Schoen et al.	2009	102	4–15	ASD: 38; SMD: 31; TD: 33^*∗∗∗*^
Su et al.	2010	31	4–8	SMD: 14; TD: 17^*∗∗∗*^
Woodard et al.	2012	16	2-3	ASD: 8; TD: 8^*∗*^

*Note*. ASD: autism spectrum disorder; TD: typically developing; TD: typically developing with atypical sensory behaviour; ADHD: attention-deficit, hyperactivity disorder; ADHDt: attention-deficit, hyperactivity disorder with typical sensory behaviour; ADHDs: attention-deficit, hyperactivity disorder with atypical sensory behaviour; SMD: sensory modulation disorder. ^*∗*^Did not assess for specific atypical sensory behaviour (i.e., overresponsivity, underresponsivity, and seeking/craving). ^*∗∗*^Reported and assessed specifically on sensory overresponsivity only. ^*∗∗∗*^Assessed for sensory modulation disorder but did not report on specific atypical sensory behavior. ^*∗∗∗∗*^Classified sensory modulation disorder based severity of atypical sensory behavioural symptoms (mild, moderate, and severe).

**(b):** 

Author	Year	Behavioural measure^*∗*^	Physiological measure		
			Autonomic branch	Autonomic measure	Specific measure
Chang et al.	2012	Sensory Processing Measure- Home Form	SNS	Skin conductance	Amplitude, magnitude, onset latency, habituation

Daluwatte et al.	2015	Sensory Profile	Not specific^*∗∗*^	Pupillary light reflex (PLR)	Diameter, PLR latency, constriction time, redilation time, constriction amplitude

Lane et al.	2010	Sensory Overresponsivity Scale	SNS	Electrodermal response	Tonic, nonspecific response, magnitude of orienting response, mean response

Mangeot et al.	2001	Short Sensory Profile	SNS	Electrodermal response	Magnitude of largest response

Matsushima et al.	2016	Short Sensory Profile- Japanese	PNS	Heart rate variability	High-frequency (0.05–1.04 Hz)

McCormick et al.	2014	Short Sensory Profile	SNS	Electrodermal activity	Tonic, magnitude of response, no response

McIntosh et al.	1999	Short Sensory Profile	SNS	Electrodermal response	Mean magnitude, amplitude of largest response, no. of responses

Miller et al.	2012	Short Sensory Profile	SNS	Electrodermal response	Mean peak magnitude of response, amplitude of largest peak

Schaaf et al.	2003	Short Sensory Profile	PNS	Cardiac vagal tone	(Frequency not specified)

Schaaf et al.	2010	Short Sensory Profile	PNS	Cardiac vagal tone	0.25–1.04 Hz

Schaaf et al.	2015	Short Sensory Profile	PNS	Respiratory sinus arrhythmia	0.15–0.50 Hz
			SNS	Preejection period	Q-wave

Schoen et al.	2009	Short Sensory Profile	SNS	Electrodermal activity	Skin conductance level, orienting response, amplitude, magnitude

Su et al.	2010	Sensory Profile; Evaluation of Sensory Processing	SNS	Electrodermal response	Mean magnitude

Woodard et al.	2012	Sensory Profile-Infant/Toddler	Not specific^*∗∗*^	Heart rate reactivity	Heart rate

*Note*. PNS: parasympathetic nervous system; SNS: sympathetic nervous system.^*∗*^Parent-/caregiver-reported measures; ^*∗∗*^from an ANS dually innervated organ.

**(c):** 

Author	Year	Procedures	
		Laboratory Paradigm	Conditions
Chang et al.	2012	Sensory Challenge Protocol	Rest, Stimulation, Recovery
Daluwatte et al.	2015	PLR Stimulation	Rest, Stimulation, Recovery
Lane et al.	2010	Sensory Challenge Protocol	Rest, Stimulation, Recovery
Mangeot et al.	2001	Sensory Challenge Protocol	Stimulation
Matsushima et al.	2016	Tactile and Auditory Sensory Paradigm	Rest, Stimulation, Recovery
McCormick et al.	2014	Sensory Probe Paradigm	Rest, Stimulation, Recovery
McIntosh et al.	1999	Sensory Challenge Protocol	Stimulation
Miller et al.	2012	Sensory Challenge Protocol	Stimulation
Schaaf et al.	2003	Sensory Challenge Protocol	Rest, Stimulation
Schaaf et al.	2010	Sensory Challenge Protocol	Rest, Stimulation, Recovery
Schaaf et al.	2015	Sensory Challenge Protocol	Rest, Stimulation, Recovery
Schoen et al.	2009	Sensory Challenge Protocol	Rest, Stimulation, Recovery
Su et al.	2010	Sensory Challenge Protocol	Stimulation
Woodard et al.	2012	Multi-Sensory Paradigm	Rest, Stimulation

**(d):** 

Author	Year	Sensory behaviour differences	ANS differences
Chang et al.	2012	ASD has significantly higher sensory behavioural problems compared to TD	Stronger SNS activation at rest and in response to auditory stimuli in ASD more TD

Daluwatte et al.	2015	Significant atypical sensory behaviour in ASD compared to TD	No significant difference in autonomic outcomes

Lane et al.	2010	Significant overresponsive sensory behaviour in ADHD compared to TD	Significantly higher EDR (SNS) in ADHD at recovery conditions

Mangeot et al.	2001	Significant sensory behavioural problems in ADHD compared to TD	Greater EDR (SNS) magnitude in stimulation conditions

Matsushima et al.	2016	ASD has significantly higher sensory behavioural problems compared to TD	Significantly lower HRV-HF (PNS) at rest in ASD compared to TD

McCormick et al.	2014	Significantly different sensory behaviour between ASD and TD	No significant difference in any measure of EDA

McIntosh et al.	1999	SMD was differentiated from TD based on severity of sensory behavioural problems	SMD failed to respond to sensory stimuli; more EDR and magnitude; and slower habituation, compared to TD

Miller et al.	2012	Significant group differences when compared with TD on the incidence of sensory behavioural problems	SMD has greater reactivity to sensory stimuli; SMD has different autonomic patterns compared to ADHD

Schaaf et al.	2003	SMD was differentiated from TD based on severity of sensory behavioural problems	SMD has significantly lower CVT (PNS) compared to TD

Schaaf et al.	2010	SMD was differentiated from TD based on severity of sensory behavioural problems	SMD approached significantly lower CVT (PNS) reaction compared to TD; severe SMD has significantly lower CVT at baseline and during stimulation compared to other groups

Schaaf et al.	2015	SMD was differentiated from TD based on severity of sensory behavioural problems	ASD has less variable PNS functions (less change from sensory domains) during stimulation conditions compared to TD

Schoen et al.	2009	Different patterns of sensory behaviour between ASD, SMD, and TD	ASD has lower baseline and reactivity SNS functions; SMD has higher SNS reactivity on the first stimulus presentation

Su et al.	2010	SMD has more sensory behavioural problems compared to TD	SMD has larger EDR (SNS) peak amplitude

Woodard et al.	2012	ASD has more hypersensitive and less hyposensitive sensory behavioural problems	ASD has more hypersensitive and less hyposensitive autonomic patterns of responses to sensory stimulus

*Note*. HRV-HF: heart rate variability-high frequency; EDR: electrodermal response; EDA: electrodermal activity; CVT: cardiac vagal tone.

**(e):** 

Author	Year	Correlation summary	
		Clinical group	Typical group
Chang et al.	2012	Total score and SC baseline^*∗*^ (*r* = 0.45) Total score and tone amplitude^*∗*^ (*r* = 0.56)	*No significant correlations* Total score and SC baseline (*r* = −0.08) Total score and tone amplitude (*r* = −0.32)

Daluwatte et al.	2015	Sensory behaviour and PLR intensity at 872.1^*∗∗*^ (*r* = 0.32) and 8721.1^*∗∗*^ (*r* = 0.39); and PLR adaptation^*∗*^ (*r* = 0.23)	*No significant correlations* Sensory behaviour and PLR intensity at 872.1 (*r* = −0.11) and 8721.1 (*r* = −0.06); and PLR adaptation (*r* = −0.01)

Lane et al.	2010		

Mangeot et al.	2001		

Matsushima et al.	2016	Sensory behaviour and resting state HRV-HF^*∗*^ (*r* = 0.38)	*No significant correlations* Sensory behaviour and resting state HRV-HF (*r* = −0.17)

McCormick et al.	2014	*No significant correlations* (no data provided)	*No significant correlations* (no data provided)

McIntosh et al.	1999		

Miller et al.	2012		

Schaaf et al.	2003		

Schaaf et al.	2010		

Schaaf et al.	2015		

Schoen et al.	2009	*No significant correlations* (no data provided)	*No significant correlations* (no data provided)

Su et al.	2010		

Woodard et al.	2012	Total sensory behaviour and HR at stimulus presentation^*∗*^ (*r* = 0.205)	*No significant correlations* Total sensory behaviour and HR at stimulus presentation (*r* = 0.132)

*Note*. ^*∗*^Sig at *p* = 0.05; ^*∗∗*^Sig at *p* = 0.01; SC: skin conductance; PLR: pupillary light reflex; HRV-HF: heart rate variability-high frequency; HR: heart rate.

**Table 2:** Summary of variations in the Sensory Challenge Protocol (SCP) across the reviewed studies.

Author	Year	Sensory Challenge Protocol description	Presentation
Chang et al.	2012	Modification of the original SCP: (1) addition of standard tone at 84 dB; (2) siren at 78 dB	6 stimuli × 10 trials × 3 s at pseudorandom 12–17 s ISI
Lane et al.	2010	Modification of the original SCP: (1) used a classic steady tone at 78 dB; (2) fire engine at 84 dB	6 stimuli × 10 trials × 3 s at variable 10–17 s ISI
Mangeot et al.	2001	Used the original SCP	5 stimuli × 10 trials × 3 s at pseudorandom 15 or 19 s ISI × 20 s between sensory domains
McIntosh et al.	1999	Used the original SCP	5 stimuli × 10 trials × 3 s at pseudorandom 15 or 19 s ISI × 20 s between sensory domains
Miller et al.	2012	Used the original SCP	5 stimuli × 10 trials × 3 s at pseudorandom 15–19 s ISI × 20 s between sensory domains
Schaaf et al.	2003	Used the original SCP	5 stimuli × 10 trials × 3 s at pseudorandom 12–17 s ISI × 20 s between sensory domains
Schaaf et al.	2010	Modification of the original SCP: (1) addition of a tone at 84 dB; (2) siren at 78 dB; (3) 2-minute auditory tone at 75 dB	7 stimuli × 10 trials × 3 s at pseudorandom 12–17 s ISI × 20 s between sensory domains
Schaaf et al.	2015	Modification of the original SCP: (1) addition of a tone at 84 dB; (2) siren at 78 dB; (3) 2-minute auditory tone at 75 dB	7 stimuli × 10 trials × 3 s at pseudorandom 12–17 s ISI × 20 s between sensory domains
Schoen et al.	2009	Modification of the original SCP: (1) used a classic steady tone at 78 dB; (2) fire engine at 84 dB	6 stimuli × 10 trials × 3 s at variable 10–17 s ISI
Su et al.	2010	Used the original SCP	6 stimuli × 8 trials × 3 s at pseudorandom 12–17 s ISI

*Note*. ISI = interstimulus interval.

**Table 3:** Summary of the Sensory Challenge Protocol [52].

Features	Olfactory	Auditory	Visual	Tactile	Vestibular
Stimulus	Wintergreen oil contained in a small vial with a ball of cotton; kept at 1.25 cm deep.	Fire engine siren played at 95 dB.	20-watt strobe light flashing at 10 Hz.	Finger puppet with a 5 cm feather attached distally	Chair placed on top of a tilted board supported by a 10 cm cube placed at each corner. Platform is a 75 cm square plywood board attached to a rotation board

Procedure	Experimenter covered a vial with his thumb until about to be presented. The vial was placed 2.5 cm away from child's nose moving from left to right.	Child listened to the tape-recorded sound.	Light was placed 60 cm away from the child, slightly below eye level and flashed continuously within the trial time window.	A feather was placed gently over the child's right ear canal and moved towards the left ear canal, passing along the bottom of the chin.	The chair was tipped backwards slowly and smoothly at an angle of 30° and placed upright afterwards.

Number of trials	10 trials				

Trial time	3 s/trial				

ISI	15 or 19 s				

Order of presentation	Olfactory → Auditory → Visual → Tactile → Vestibular				

Intersensory domain interval	20 s				

Setting	The laboratory was designed to look like a spaceship, with the paradigm designed to resemble a “spaceship trip.” The child was seated in front of a spaceship console with a 13-inch monitor screen and strobe light placed 60 cm away, at eye level. While preparing the experiment, the child watched a spaceship-themed video clip that was chosen to be nonstimulating but entertaining.				
